# Valproic acid enhances the efficacy of radiation therapy by protecting normal hippocampal neurons and sensitizing malignant glioblastoma cells

**DOI:** 10.18632/oncotarget.5253

**Published:** 2015-09-16

**Authors:** Dinesh Thotala, Rowan M. Karvas, John A. Engelbach, Joel R. Garbow, Andrew N. Hallahan, Todd A. DeWees, Andrei Laszlo, Dennis E. Hallahan

**Affiliations:** ^1^ Department of Radiation Oncology, Washington University in St. Louis, Missouri, USA; ^2^ School of Medicine, Washington University in St. Louis, Missouri, USA; ^3^ Mallinckrodt Institute of Radiology, Washington University in St. Louis, Missouri, USA; ^4^ Siteman Cancer Center, Washington University in St. Louis, Missouri, USA; ^5^ Hope Center, Washington University in St. Louis, Missouri, USA

**Keywords:** valproic acid (VPA), neuroprotection, histone deacetylase (HDAC), radioprotection, cancer therapy

## Abstract

Neurocognitive deficits are serious sequelae that follow cranial irradiation used to treat patients with medulloblastoma and other brain neoplasms. Cranial irradiation causes apoptosis in the subgranular zone of the hippocampus leading to cognitive deficits. Valproic acid (VPA) treatment protected hippocampal neurons from radiation-induced damage in both cell culture and animal models. Radioprotection was observed in VPA-treated neuronal cells compared to cells treated with radiation alone. This protection is specific to normal neuronal cells and did not extend to cancer cells. In fact, VPA acted as a radiosensitizer in brain cancer cells. VPA treatment induced cell cycle arrest in cancer cells but not in normal neuronal cells. The level of anti-apoptotic protein Bcl-2 was increased and the pro-apoptotic protein Bax was reduced in VPA treated normal cells. VPA inhibited the activities of histone deacetylase (HDAC) and glycogen synthase kinase-3β (GSK3β), the latter of which is only inhibited in normal cells. The combination of VPA and radiation was most effective in inhibiting tumor growth in heterotopic brain tumor models. An intracranial orthotopic glioma tumor model was used to evaluate tumor growth by using dynamic contrast-enhanced magnetic resonance (DCE MRI) and mouse survival following treatment with VPA and radiation. VPA, in combination with radiation, significantly delayed tumor growth and improved mouse survival. Overall, VPA protects normal hippocampal neurons and not cancer cells from radiation-induced cytotoxicity both *in vitro* and *in vivo*. VPA treatment has the potential for attenuating neurocognitive deficits associated with cranial irradiation while enhancing the efficiency of glioma radiotherapy.

## INTRODUCTION

Radiotherapy, the clinical application of ionizing radiation, is a crucial treatment option in modern cancer therapy in addition to surgery and systemic therapy. This is corroborated by the fact that more than 60% of all cancer patients receive radiotherapy [1, 2]. Cranial irradiation is commonly used for the treatment of neoplasms involving the central nervous system. Radiotherapy can have negative sequelae of acute neurocognitive deficits, especially in the pediatric population [3–5]. The pathogenesis of radiation-induced neurocognitive deficits involves apoptosis of neuroproliferative cells in the subgranular zone of the hippocampus, a region in the brain vital for learning and memory [6–11]. Several studies have demonstrated a steep, long-term decline in subgranular neurogenesis in the dentate gyrus following radiation exposure [11–15] and direct irradiation of the hippocampus has been shown to result in pronounced cognitive deficits [16]. The cognitive deficits following hippocampal irradiation include deficits of learning, memory, and spatial processing [17, 18]. Other areas of the cerebrum appear to be less sensitive to the effects of radiation [19]. Although at present there is no pharmacological prophylaxis available for the prevention of radiation-induced neurocognitive deficits, the drugs memantine and minocycline are promising novel agents. Memantine attenuated cognitive function decline after whole brain radiotherapy (WBRT) in patients with brain metastases [20]. Minocycline ameliorated cognitive impairment induced by WBRT in animal models [21].

Valproic acid (VPA) is a short branched–chain carboxylic acid that is an FDA approved anti-seizure and antidepressant drug that is well tolerated and whose toxicity profile has been extensively characterized [22]. VPA has a neuroprotective effect in the setting of various neurological insults, including ibetinoic acid [23], glutamate toxicity [24, 25], intracerebral hemorrhage [26], ischemia [27, 28], malonate toxicity [29], and oxidative stress [30]. The inhibition of histone deacetylase (HDAC) activity by VPA is thought to be involved in such neuroprotection [24–27, 31–34]. VPA inhibits both class I and II HDACs with resultant hyperacetylation of histone H3 and H4 [35–39]. In addition to HDAC inhibition, VPA has been shown to have effects on the Akt/GSK3β pathway, an observation that provides a novel facet of mechanisms involved in the neuroprotective effects of VPA [40]. Other pathways have also been proposed, including the induction of alpha-synuclein [24], heat shock protein 70 [34], brain-derived neurotrophic factor [41], and modulation of the JNK pathway [42].

In addition to protecting healthy neurons, treatment with VPA can selectively kill various cancer cell lines including glioblastoma [43–45], erythroleukemic cells [43], colon cancer cells [46] and prostate cancer cells [47]. The preclinical data of VPA on 19 different types of solid cancers including glioma have been summarized [48, 49]. The mechanism of sensitization is proposed to involve inhibition of HDAC leading to interference with the repair of double strand DNA breaks [50–53]. VPA is also known to inhibit cell growth in cancer cells by inducing apoptosis and cell cycle arrest [54, 55].

GBM patients treated with VPA as an anticonvulsant agent during treatment with radiochemotherapy have recently drawn attention because of better outcomes of the treatment, reviewed in [56]. Combined therapy with VPA produced a 3-month longer overall survival (OS) as compared with carbamazepine in patients with GBM [57]. Several recent studies have found improved OS in both children and adults, ranging from 3–6 months by the inclusion of VPA [48, 58–61]. It has been speculated that the HDAC inhibitory properties of VPA could mediate the prolonged survival derived from radio-chemotherapy seen in GBM patients [62].

Whole genome expression monitored by microarray analysis of primary tumors of patients treated with VPA showed significant up-regulation of hundreds of genes belonging to multiple pathways. These include ribosomal proteins, oxidative phosphorylation, MAPK signaling, focal adhesion, cell cycle, antigen processing and presentation, proteasome, apoptosis, PI3K, Wnt signaling, calcium signaling, TGF-beta signaling, and ubiquitin-mediated proteolysis among others [49].

In the present study, we found that VPA protected the normal hippocampus from radiation-induced damage, while sensitizing malignant gliomas to radiation. We analyzed the anti-apoptotic effects of VPA administration *in vitro* and *in vivo* and characterized the changes in intracellular signaling and protein expression induced by administration of VPA prior to radiation. We also determined the radiosensitizing effect of VPA in glioblastoma cell lines, and its effects on tumor growth delay and survival of intracranial glioma-bearing mice using dynamic contrast enhanced magnetic resonance imaging, DCE MRI.

## RESULTS

### VPA treatment protects hippocampal neurons from radiation-induced apoptosis *in vivo*

**Figure 1:**
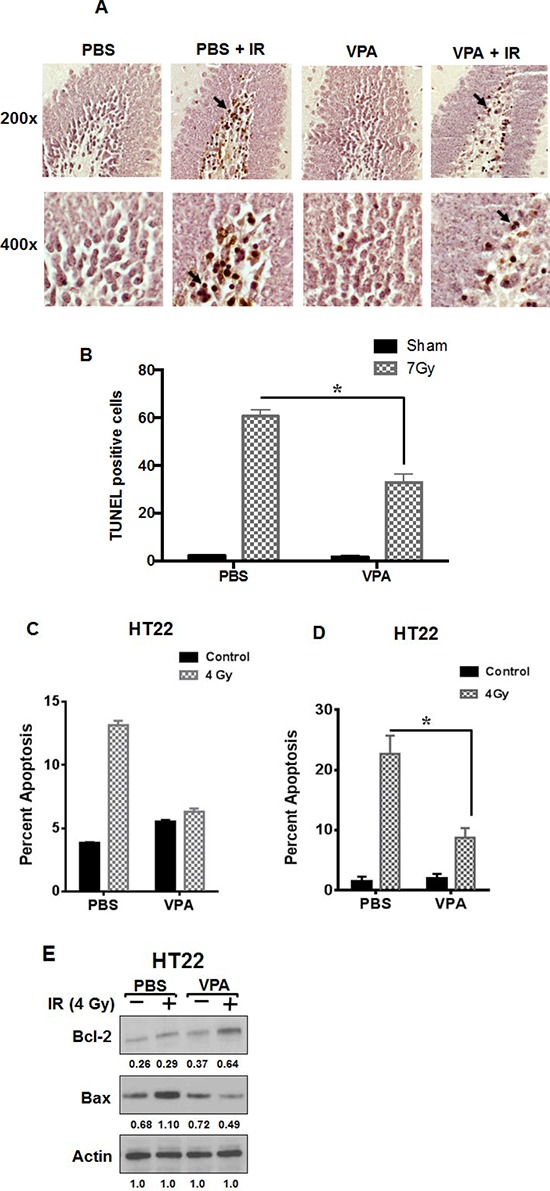
VPA treatment protects hippocampal neurons from radiation-induced apoptosis *in vivo* and modulates the expression of apoptotic signaling proteins *in vitro* **A.** One-week-old C57BL/6 mice pups were treated with daily i.p. injections of VPA (300 mg/kg) or PBS for 7 days. One hour after the last VPA treatment, the pups heads were irradiated with 7 Gy while the rest of the body was shielded with lead. Twenty-four hours later, the animals were sacrificed and brains were fixed and coronally sectioned. Sections that contained hippocampus were stained with TUNEL. Shown are representative photographs of mouse hippocampus. The arrows indicate examples of TUNEL positive cells (TPC). **B.** Eight HPF at 200x magnification were evaluated and TPC were counted for each experimental group. Shown is the average number of TPC per HPF for each radiation dose group (SD of three independent experiments, *P < 0.05). **C.** HT22 cells were treated with PBS or 0.6 mM VPA for 7 days prior to irradiation with 4 Gy. 24 h after irradiation, cells were stained with Annexin V-APC/propidium iodide and analyzed by flow cytometry; *P < 0.05 **D.** Cells were fixed and stained with DAPI, and apoptotic cells were counted in eight randomly selected HPF at 200X magnification. Shown are bar graphs of the average percent of apoptotic cells for each treatment with SD from three experiments; *P < 0.05. **E.** HT22 cells were treated with PBS or 0.6 mM VPA for 7 days prior to irradiation with 4 Gy. Whole cell extracts were immunobloted to determine the levels of Bax and Bcl-2. Actin was used to normalize the protein loading in each lane. Densitometry values representing the ratio of the various proteins normalized actin is indicated below each immunoblot.

To determine whether VPA regulates cell survival and apoptosis in the subgranular zone of the hippocampus after irradiation, we pretreated 1-week-old mice with 300 mg/kg of VPA for 7 days prior to cranial irradiation with 7Gy. Typically, glioblastoma patients are treated with fractionated focal irradiation delivered as daily fractions of 2 Gy given five days per week for 6 weeks, for a total of 60 Gy [63]. We have shown that treatment with 7 Gy is in the steep part of the sigmoidal dose response curve for radiation-induced apoptosis in the mouse hippocampus [64] and hence we used 7 Gy for our studies. Apoptosis within the subgranular zone was determined by staining the hippocampal sections with a TUNEL kit, and the TUNEL positive cells (TPC) were counted (Figs. 1A & 1B). Three sections from each of three different mice in each treatment (a total of 9 sections per treatment) were analyzed. Mice irradiated with 7Gy had increased apoptosis compared to untreated mice. Mice treated with VPA prior to 7Gy irradiation had significantly fewer TPC compared to mice irradiated with 7 Gy (*P* < 0.001; Fig. 1B), indicating that VPA treatment protected the mouse hippocampus from radiation-induced apoptosis.

### VPA treatment attenuates radiation-induced apoptosis in HT22 cells

We monitored radiation-induced apoptosis *in vitro* by staining irradiated normal hippocampal HT22 cells with Annexin V-APC and propidium iodide. The stained cells were analyzed by flow cytometry after various experimental treatments (Fig. 1C). Cells pre-treated with VPA prior to 4Gy irradiation had significantly less apoptotic cells (12% annexin V positive: *P* = 0.002), than cells treated with PBS alone (50%; Fig. 1C). To further confirm these results, we monitored the nuclear morphology of irradiated cells using DAPI staining (Supplemental Fig. 1, Fig. 1D). Pre-treatment of irradiated HT22 cells with VPA led to a protective effect, with a reduced number of apoptotic cells (15%) compared to 35% in PBS-pretreated cells (*P* < 0.001; Fig. 1D). We did observe a slight increased apoptosis when cells were treated with VPA when compared to PBS; this however was not statistically significant. Treatment of HT22 cells with VPA led to decreased levels of the pro-apoptotic protein BAX and increased levels the anti-apoptotic protein Bcl-2 (Fig. 1E), which is consistent with the results obtained using the other endpoints for apoptosis described above. However, we did not detect any PARP cleavage in irradiated HT22 cells as has been reported before (Supplementary Fig. 2) [65].

### VPA treatment reduces GL261 cell survival

**Figure 2:**
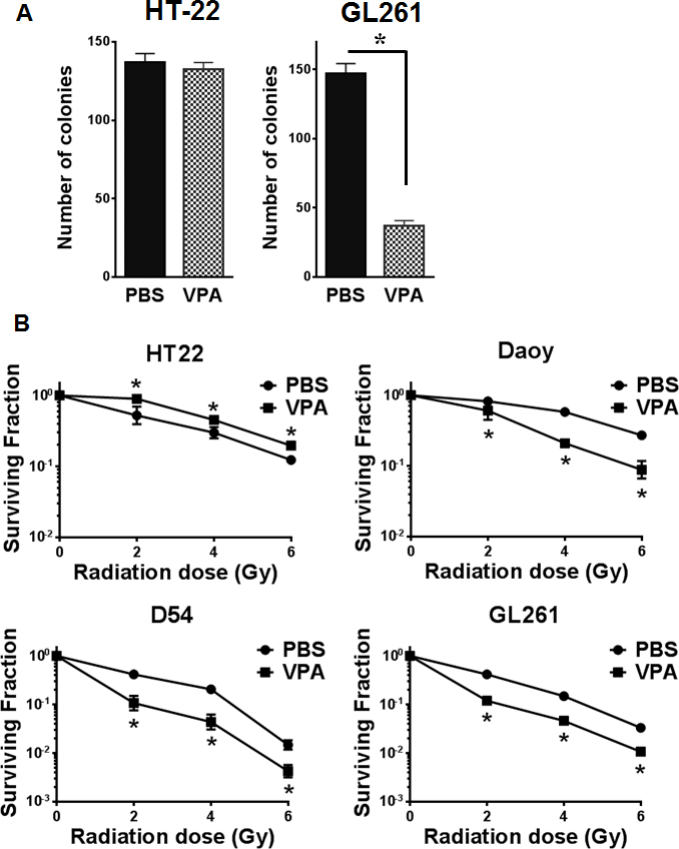

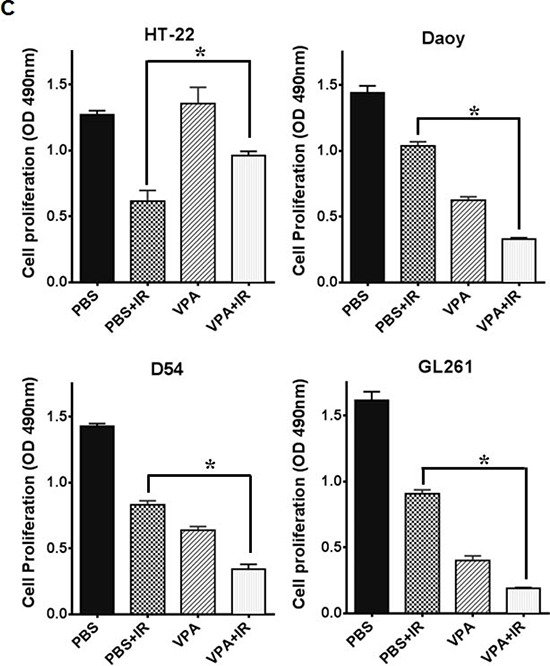
VPA acts as a radioprotector in normal cells and radiosensitizer in cancer cells **A.** Equal numbers of HT22 or GL261 cells were plated for colony formation assay after treating with PBS or 0.6 mM VPA for 7 days. Plates were stained with 1% methylene blue after 10 days and colonies were counted. Shown are bar graphs depicting the number of colonies for each treatment with SD from three experiments; *P < 0.05. **B.** HT22, Daoy, D54 and GL261 cell were treated with PBS (●) or 0.6 mM Valproic acid (■) for 7 days followed by irradiation with 0, 2, 4, 6 or 8 Gy and plated for clonogenic survival assay. Shown are the surviving fractions and the SD from three experiments; *P < 0.05. **C.** Equal numbers of HT22, Daoy, D54 and GL261 cells were plated in 96-well plates after treatment with PBS or 0.6 mM VPA for 7 days and then irradiated with 4Gy. After 96 h, the cell viability was determined using a colorimetric cell proliferation assay. Shown are the absorbances at 490 nm; *P < 0.05.

To determine the effect of VPA treatment on cell viability and survival of hippocampus-derived HT22 cells and glioblastoma GL261 cells, we performed a colony formation assay. Cells were treated with 0.6 mM VPA or PBS for 7 days and equal numbers of cells were plated to determine plating efficiency. There was no significant difference in the numbers of colonies from HT22 cells treated with VPA (*P* = 0.398) compared to PBS treated cells (Fig. 2A). However, treatment of GL261 cells with VPA led to a significant decrease in colony formation (*P* = <0.001) compared to PBS control (Fig. 2A).

### VPA treatment protects HT22 cells from radiation while sensitizing Daoy, D54 and GL261 cells

To determine the effect of VPA treatment on the cellular response to radiation, we performed clonogenic cell survival assays. Pretreatment of hippocampus-derived HT22 cells with 0.6 mM VPA for 7 days significantly abrogated radiation-induced cell killing (2Gy *P* = 0.040, 4Gy *P* = 0.016, 6Gy *P* = 0.060, 8Gy *P* = 0.002) as compared to cells treated with radiation alone with a DMF_10_ of 0.87 (Fig. 2B). However, pretreatment of Daoy (human medulloblastoma), D54 (human glioma) and GL261(mouse glioma) cells with 0.6 mM VPA for 7 days prior to irradiation led to significant radiosensitization with DMF_10_ of 2.25, 1.49, and 2.31 for Daoy, D54 and GL261 cells, respectively (Fig 2B). These results indicate that VPA treatment protects normal hippocampal neuronal cells (HT22) from radiation induced cell killing, while radiosensitizing medulloblastoma cells (Daoy) and GBM cells (D54 & GL261).

### VPA treatment inhibits Daoy, D54 and GL261 cell proliferation

Having observed radiosensitization of cancer cells (Daoy, D54 and GL261) and radioprotection of normal cells (HT22) after VPA treatment in clonogenic assays, we wanted to ascertain if this was due to alterations in cell proliferation. HT22, Daoy, D54 and GL261 cells were treated with PBS or VPA (0.6 mM) for 7 days and then equal numbers of treated cells (of each cell line) were plated in 96-well plates. The plates were then incubated for 96 h, and then 20 μL of cell proliferation assay reagent (Promega) was added. HT22 cells treated with VPA did not show any differences in proliferation compared to PBS treated cells (*P* = 0.065; Fig. 2C). However, treatment with VPA led to reduced cell proliferation in Daoy cells (*P* = <0.002), D54 cells (*P* = <0.001) and GL261cells (*P* = <0.001; Fig. 2C). Thus the anti-proliferative effect of VPA treatment was observed only in cancer cells (Daoy D54 and GL261) and not in normal neuronal cells (HT22).

### VPA treatment induces the accumulation of GL261 cells at G_2_/M

**Figure 3:**
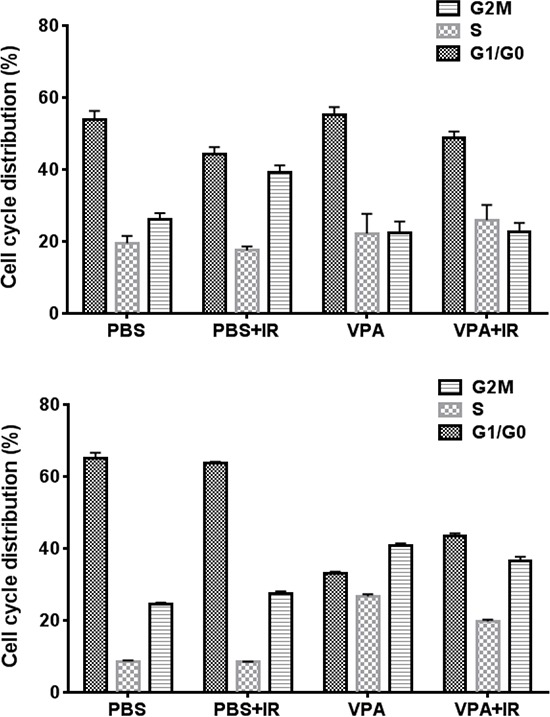
VPA treatment induces G2/M accumulation in cancer cells HT22 cells and GL261 cells were treated with PBS or 0.6 mM VPA for 7 days prior to irradiation with 4 Gy. 24 h after irradiation, cells were collected, fixed and stained with PI and cell cycle distributions were determined by flow cytometry. Shown are the bar graphs of the average change in the fraction of G_1_/G_0_, S and G_2_/M phase cells after each treatment.

Since VPA treatment led to inhibition of cell proliferation only in cancer cells, we compared the effect of VPA treatment on the cell cycle distribution of normal vs. cancer cells. HT22 and GL261 cells were treated with PBS or VPA (0.6 mM) for 7 days prior to irradiation with 4Gy. The cells were harvested 24 h post irradiation, stained with propidium iodide and analyzed by flow cytometry. There were no significant alterations of cell cycle distribution in HT22 cells treated with VPA alone, while irradiation led to the accumulation of cells in the G_2_/M phase (40%) compared to untreated PBS controls (18%; *P* = <0.001). The accumulation in G_2_/M was abrogated when cells were treated with VPA (25%) prior to irradiation (*P* = <0.001; Fig. 3). Significant accumulation in the G_2_/M phase (41%) was observed in GL261 cells treated with VPA when compared to untreated PBS control cells (24%) (*P* = <0.001). A larger fraction of G_2_/M accumulation was observed when they were treated with a combination of VPA and irradiation with 4Gy (37%) compared to irradiation alone (28%; *P* = <0.001). This increase was however not statistically significant from the G2/M accumulation induced by VPA treatment alone (40%; Fig. 3). There was no major effect in the fraction of S phase cells in either cell line after the various experimental treatments.

### VPA treatment inhibits both HDAC and GSK3β in HT22 cells

**Figure 4:**
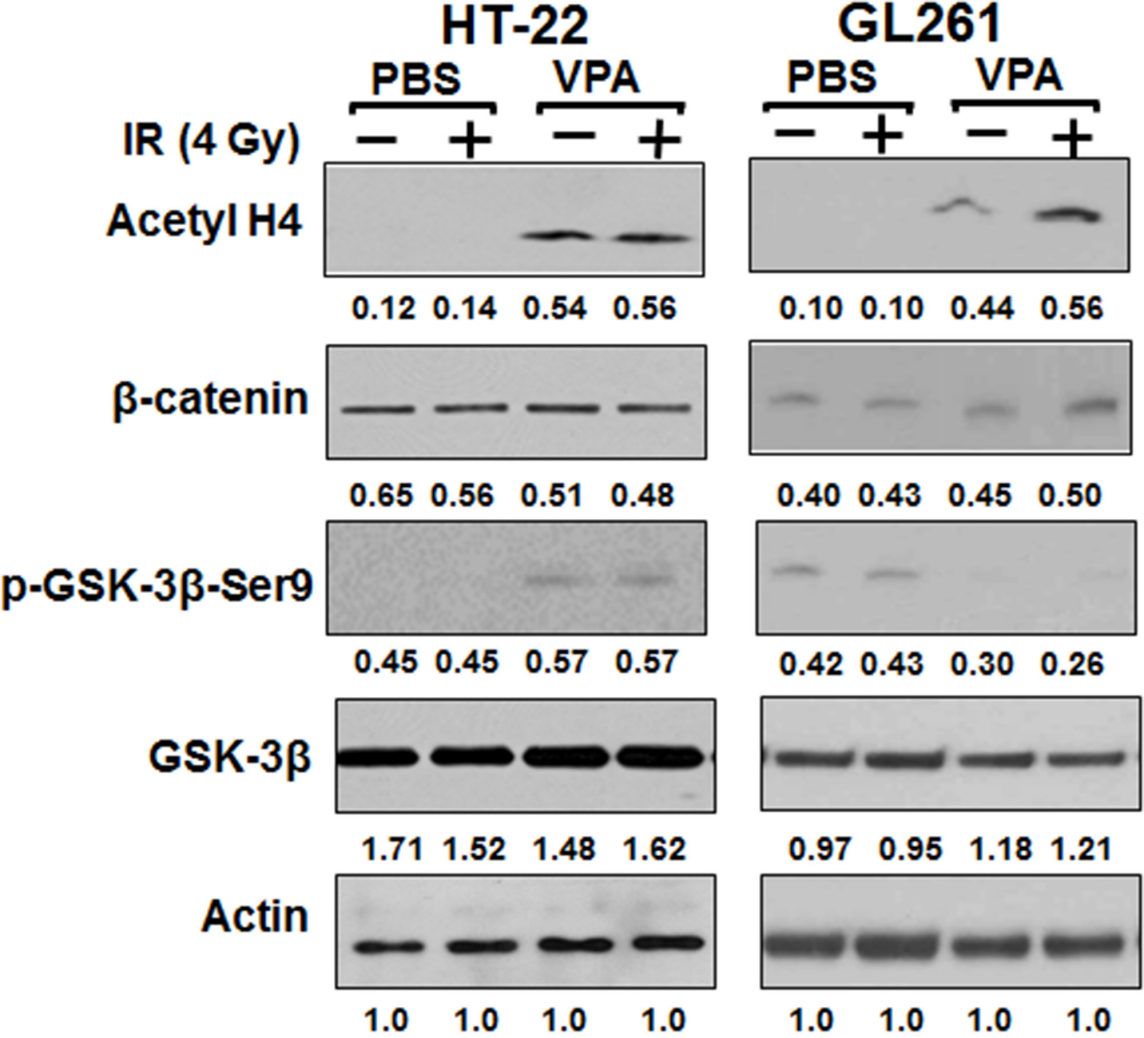
VPA inhibits both HDAC and GSK3β in normal cells, but only HDAC in cancer cells HT22 and GL261 cells were treated with 0.6 mM VPA for 7 days prior to irradiation with 4 Gy. Total cellular proteins were immunobloted using antibodies against β-catenin, p-GSK3β (Ser9), GSK3β and Acetyl H4 to determine their levels. A representative (from 3 repeats) immunoblot is shown.

VPA inhibits the activity of histone deacetylases, HDACs, most likely by binding to the catalytic center of the enzyme [35]. VPA has also been reported to inhibit the activity of glycogen synthase kinase 3 beta (GSK3β) both *in vitro* [66, 67] and *in vivo* [67]. To determine if HDAC activity was inhibited by VPA treatments of HT22 and GL261 cells we monitored the levels of acetylated histone H4. To determine if VPA treatment inhibits GSK3β in HT22 hippocampal neurons and GL261 glioblastoma cells, we monitored the phosphorylation of GSK3β at ser9 that inhibits its activity and evaluated the levels of β-catenin as a surrogate indicator of GSK3β activity. Since active GSK3β phosphorylates β-catenin that promotes its degradation, increased accumulation of β-catenin correlates with decreased activity of GSK3β. Inhibition of HDAC activity was observed in both HT22 and GL261 cells treated with 0.6 mM VPA for 7 days (Fig. 4). Interestingly, the inhibition of GSK3β by VPA treatment was observed only in HT22 cells and not in GL261cells (Fig. 4). These results indicate that VPA, in addition to inhibiting HDAC, also inhibits GSK3β in HT22 cells but not in GL261cells. Thus, the overall mechanisms of the action of VPA are different in these two cell lines. VPA treatment associated inhibition of GSK3β activity contributed to reduced radiation-induced apoptosis in HT22 cells (Fig. 1C). We have previously shown that inhibition of GSK3β either with small molecule inhibitors or knockdown led to decreased apoptosis in normal hippocampal neurons and normal epithelial cells [64, 68, 69].

### VPA treatment represses tumor growth in irradiated GL261 and D54 mouse tumor models

**Figure 5:**
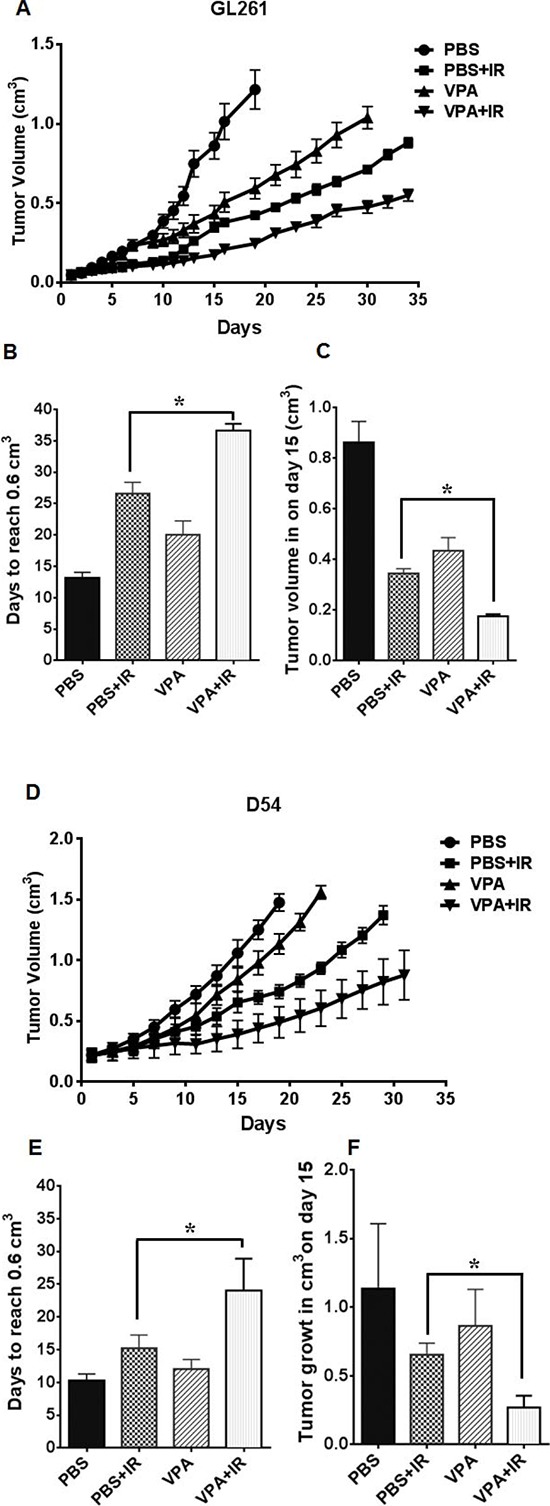
VPA delays tumor growth in irradiated mouse tumor models GL261 or D54 cells were implanted into the right flank of nude mice. Once the tumors were palpable, they were irradiated with 2 Gy for 5 consecutive days for a total of 10 Gy. Mice were treated with VPA (300 mg/Kg) or PBS 60 min prior to irradiation. Tumor volumes were measured using digital calipers. Shown are the mean tumor volumes with SD from each treatment group of eight mice **A & D,** number of days to reach a tumor volume of 0.6 cm^3^ **B & E,** *P < 0.05, mean tumor volume following various treatments on day 15 **C & F,** *P < 0.05.

To determine the *in vivo* efficacy of VPA, two heterotopic glioma models GL261 (mouse) and D54 (human) were used to generate tumor growth delay curves (Fig. 5A & 5D). One million GL261 or D54 cells were injected subcutaneously in the right hind limb of nude mice. Tumor-bearing mice were then treated with PBS alone, irradiation alone (five daily fractions of 2 Gy), VPA (300 mg/kg) alone for 5 days, or VPA (300 mg/kg) for 5 days followed by irradiation (five daily fractions of 2Gy). In studies on the effect of VPA in mice models, doses ranging from 150 mg/Kg to 400 mg/Kg have been used [49, 67, 70]. The time required to reach a tumor volume of 0.6 cm^3^ was determined (Fig. 5B & 5E). An average of 13 days (GL261) and 10.2 days (D54) was required to reach this tumor volume in PBS treated mice, 20 days (GL261) and 12 days (D54) in the VPA treated mice, 26.5 days (GL261) and 15.2 days (D54) days in irradiated mice and 36.5 days (GL261) and 24 days (D54) in mice treated with combination of VPA and irradiation. Kruskal-Wallis and Tukeys pairwise comparisons indicated a significant difference in the number of days required to reach a tumor volume of 0.6 cm^3^ between each treatment group (Figs 5B & 5E). We also analyzed the effect of the treatments on mean tumor growth at the midpoint (the 15^th^ day) of the study (Fig. 5C & 5F). The most pronounced tumor growth delay was observed in tumors receiving a combination of VPA and irradiation (0.17 cm^3^ in GL261; 0.38 cm^3^ in D54), followed by tumors treated with irradiation alone (0.34 mm^3^ in GL261; 0.65 cm^3^ in D54), followed by tumors treated with VPA alone (0.43 cm^3^ in GL261; 0.84 cm^3^ in D54) followed by untreated tumors (0.86 cm^3^ in GL261; 1.06 cm^3^ in D54). This tumor growth data from day 15 was also analyzed using Kruskal-Wallis and Tukeys pairwise comparisons. The growth of both GL261 and D54 tumors was significantly delayed in mice treated with the combination of VPA and irradiation when compared to mice receiving radiation alone (*P* = <0.001), or mice receiving VPA alone (*P* =< 0.001), or PBS treated controls (*P* = <0.001). Tumor growth was also significantly delayed in mice treated with VPA alone when compared to untreated control tumors (*P* = <0.001).

### VPA treatment in combination with irradiation reduces intracranial tumor growth

**Figure 6:**
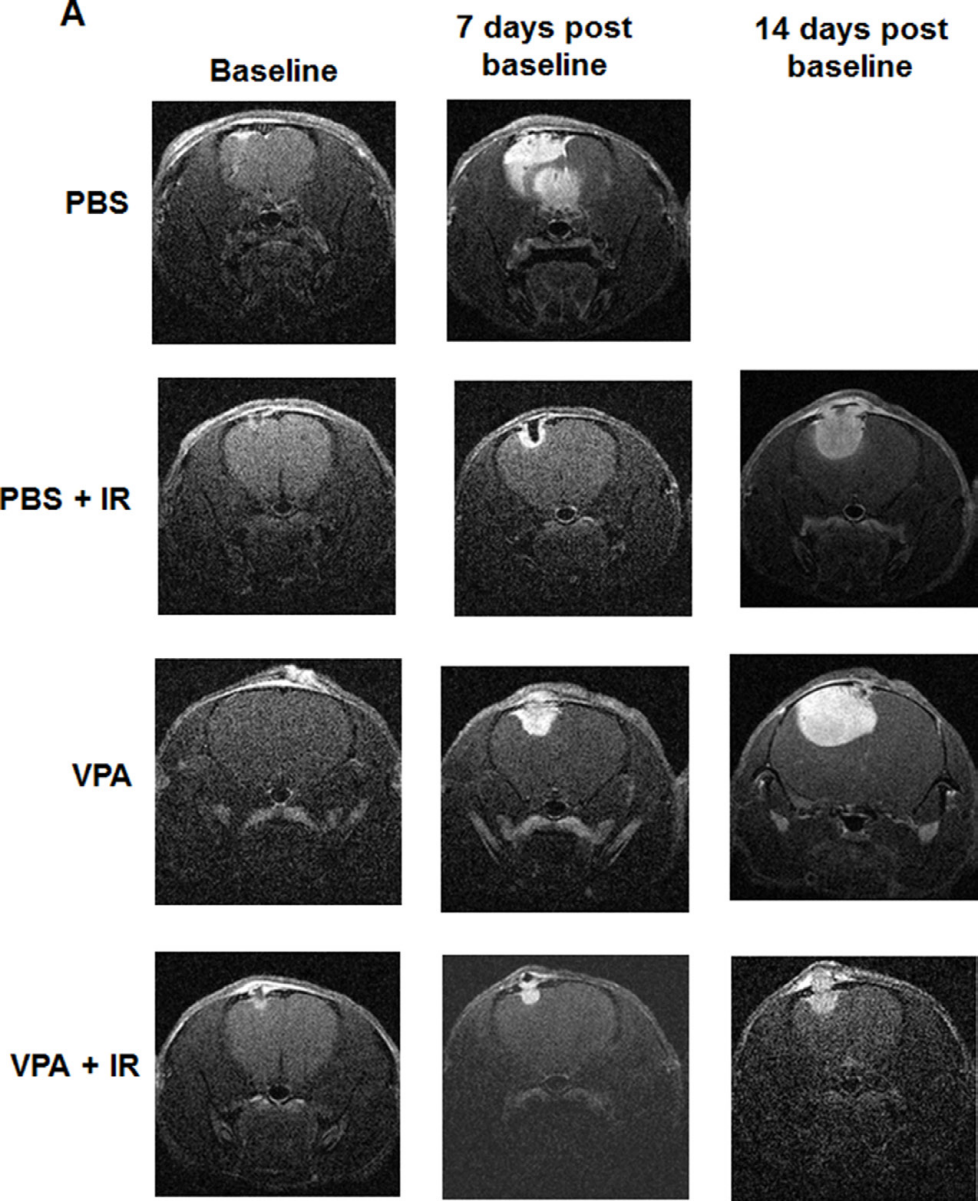

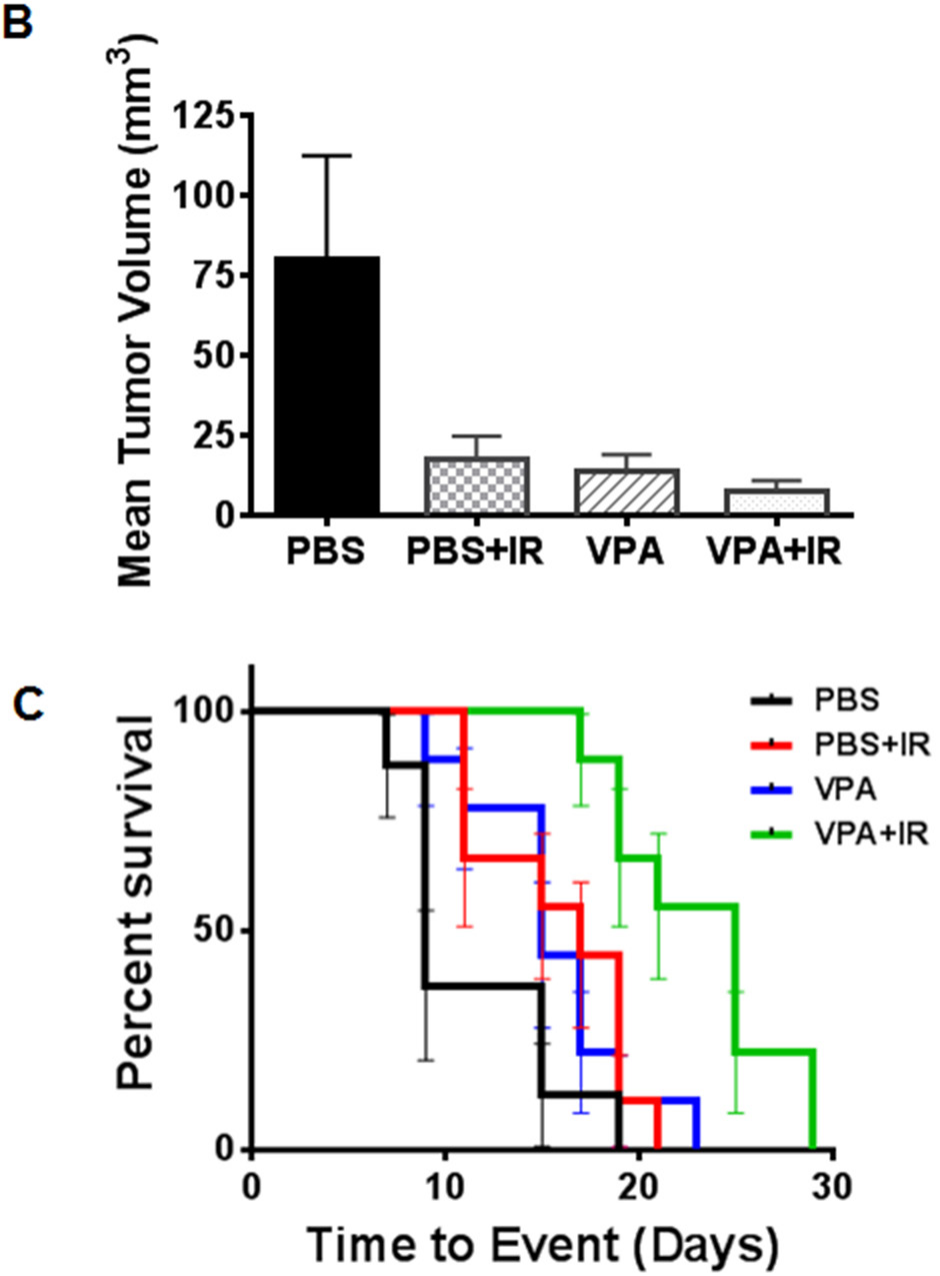
VPA in combination with irradiation improves survival by inhibiting tumor growth Nude mice were implanted intracranially with GL261 cells stably expressing luciferase. After 10 days, mice were imaged with bioluminescence imaging and then serpentine sorted into four groups of nine each. Tumors were irradiated with 2 Gy or sham for five consecutive days shielding of the non-brain areas of the body during irradiation. Mice were treated with VPA (300 mg/Kg) or PBS 60 min prior to irradiation. **A.** Representative, contrast-enhanced, T1-weighted images of mouse brain from each of the four groups (top to bottom: PBS, PBS + IR, VPA, VPA + IR) at baseline (left) and 7 days (middle) and 14 days (right) post treatment. **B.** Average, MRI-derived tumor volumes measured at seven days post-treatment. **C.** Kaplan-Meyer survival curves following various treatments of mice bearing intracranial tumors.

Magnetic resonance imaging, MRI, provides detailed information about the anatomy and morphology of brain-tumors, enabling effective diagnosis and treatment. We used dynamic contrast-enhanced magnetic resonance (DCE MRI) techniques to image the effects of VPA on tumor growth in an orthotopic intracranial GL261 mouse tumor model that stably expressed luciferase. Tumor-bearing mice were screened using bioluminescence imaging (BLI) and stratified into 4 groups having similar distribution of tumor volumes. Nine mice in each group were then treated with PBS, irradiation alone (five daily fractions of 2 Gy), VPA (300 mg/kg) alone for 5 days, or VPA (300 mg/kg) for 5 days followed by irradiation (five daily fractions of 2Gy). We obtained both contrast-enhanced T_1_-weighted and T_2_ weighted MR images for tumor growth analysis. We scanned the mice at three time points during these experiments: 1) prior to treatment (Baseline), 2) 7 days post treatment, and 3) 14 days post treatment (Fig. 6A). Regions of interest (ROI) were drawn manually around tumors using Image J software and the corresponding tumor volumes were calculated. The tumor volumes of all the treatment groups at the first baseline scan were similar, with no significant differences among the groups. In the second scan, seven days post treatment, tumor volumes for mice treated with either radiation (13.9 mm^3^) or VPA alone (17.9 mm^3^) were significantly smaller than PBS treated controls (80.3 mm^3^). The most significant reduction in volume was seen in mice treated with combination of VPA and radiation (7.4 mm^3^), indicating that such treatment reduced the growth of the glioblastoma tumor most efficiently (Fig. 6B). For the third scan (14 days post treatment), we scanned mice treated with VPA alone, irradiation alone and VPA in combination with irradiation. PBS treated control mice had to be euthanized due to tumor burden (Fig. 6A).

### VPA treatment in combination with irradiation improves survival by inhibiting tumor growth

To determine the effects of VPA on the survival of orthotopic tumor bearing mice, we used an intracranial GL261 mouse glioblastoma model stably expressing luciferase. Tumor-bearing mice were screened using bioluminescence imaging, (BLI) and stratified into four groups of nine mice each having similar distribution of tumor volumes. The mice in each group were then treated with PBS alone, radiation alone (five daily fractions of 2 Gy), VPA (300 mg/kg) alone for 5 days, or VPA (300 mg/kg) for 5 days followed by radiation (five daily fractions of 2Gy). We then analyzed the effect of the treatments on percent survival at the midpoint of the study (the 15^th^ day). Percent survival was 13% for PBS treated mice, 55% for mice treated with radiation and 44% for mice treated with VPA alone. In mice treated with VPA combined with radiation, 15-day survival was 100% (Fig. 6C). Using a Cox Proportional Hazard regression model we found that the mice receiving the combined treatment with VPA and radiation showed statistically significant increased survival when compared to PBS treated mice (*P* = 0.002), mice treated with radiation alone (*P* = 0.0130) or mice treated with VPA alone (*P* = 0.0140). The survival assay results indicate that treatment of mice with a combination of VPA and radiation was most effective (Fig. 6C).

## DISCUSSION

Radiation therapy is an effective treatment for children with medulloblastoma and some forms of leukemia. Cranial irradiation, however, results in memory and learning deficits in children [71]. The age dependence of radiation-induced brain injury has led to delaying radiotherapy in infants [72]. Treatment of infants and children with low doses of radiation to the brain often leads to long lasting neurocognitive deficits that can become permanent [73, 74]. These neurocognitive deficits manifest as declines in IQ and higher unemployment rates as compared to children treated with chemotherapy alone [75, 76]. It has been proposed that the hippocampal neuron progenitor cells are the most affected, leading to the radiation-induced neurocognitive decline [77, 78]. Radiation-induced cognitive deficits involve apoptosis in the sub-granular region of the hippocampus and the subsequent diminished neurogenesis [78, 79]. The efficacy of radiotherapy can be improved by the use of radioprotectors that prevent radiation damage to normal tissues [64, 68, 80, 81]. The efficacy of radiotherapy can also be improved by use of chemical agents that specifically sensitize cancer cells to radiation [82, 83].

In the present study, mouse hippocampus and hippocampus-derived cells (HT22) pretreated with VPA were protected from radiation damage. However, such radioprotection was not observed in the brain cancer cell lines (Daoy, D54 and GL261; Fig. 2B & 2C), or in the GL261 and D54 tumor models (Fig. 5). The selective protection of hippocampal neurons could be due to modulations of radiation-induced pro-apoptotic signaling by VPA (Fig 1E). In contrast to hippocampal neurons in the sub-granular zone, cancer cells typically do not undergo apoptosis in response to ionizing radiation. Therapeutic radiation kills most tumor cells by post-mitotic cell death through mitotic catastrophe [1, 83]. Hence this differential response to radiation provides a means to improve the therapeutic effect of cranial irradiation.

Following treatment of both normal hippocampal cells (HT22) and glioblastoma cells (GL261) with VPA, we found increased levels of acetylated histone H4, indicating that the activity of HDACs was inhibited in both normal and cancer cells (Fig 4). Several phenotypes associated with the effects of VPA are brought about by its action as an inhibitor of HDACs. These include its cytotoxicity and perturbation on the DNA damage repair machinery [22, 49]. Although VPA inhibits HDAC in both normal and cancer cells, it selectively kills cancer cells [84, 85]. Several mechanisms have been proposed to explain the preferential selectivity killing of cancer cells by VPA and other HDAC inhibitors. None of the proposed mechanism so far can fully account for this generalized effect, found in multiple cell lines and tumor types irrespective of the different cell line specific control of gene transcription [84, 85]. It has been shown that VPA induces a specific type of DNA damage that can be repaired in normal cells but not in cancer cells [86]. The nature of such DNA damage remains to be elucidated. There is evidence that HDAC inhibitors lower the cell's capacity to repair radiation-induced DNA damage. Such perturbations occur both at the level of DNA damage signaling and the major DNA repair pathways like NHEJ and HR [85, 87]. VPA and other HDAC inhibitors have been shown to decrease several key players in the repair of DNA DSB in cancer cells, at the levels of protein and/or mRNA as well as prolonged radiation-induced repair protein foci. These include Ku70, Ku80, DNA PK, ligase IV, XRCC4, RAD 51, RAD 50, ATM, γ-H2AX, BRCA1, BRCA2, and 53 BP1 [46, 87]. These alterations have been reported in cancer cells but not in normal cells. The participation of the acetylation of non-histone proteins in the increased radiosensitivity of HDAC treated cancer cells has been postulated, but remains to be demonstrated [1, 84, 88]

We found that VPA protected normal cells from radiation damage, which was in part due to the attenuation of radiation-induced apoptosis (Fig. 1A–1D; 2B). Such attenuation was associated with decreased levels of Bax, a proapoptotic protein and increased levels of Bcl-2, an antiapoptotic protein (Fig. 1E). Whether the attenuation of radiation-induced apoptosis in the hippocampus (Figs. 1A & 1B) is also associated with alterations in the levels of these proteins remains to be determined.

We observed an increase in the phosphorylation of GSK3β (ser9) and stabilization of β-catenin, indicating the inhibition of the GSK3β activity by VPA treatment, but only in normal and not in cancer cells (Fig. 4). Previously we have shown that inhibition of GSK3β either with small molecule inhibitor or knockdown protected the hippocampal neurons from radiation-induced apoptosis and increased clonogenic survival [64, 89]. Further, we have shown that GSK 3β inhibition enhances the repair of DNA DSB in irradiated normal hippocampal neurons (HT22) but not in glioma cells (GL261 and D54). This effect correlated with increased efficacy of end rejoining by the NHEJ DSB repair pathway [89]. Hence, we speculate that the neuroprotection of hippocampal neurons *in vivo* involves such enhancement of the efficacy of the NHEJ by GSK3β. The lack of inhibition of VPA-mediated GSK3β in glioma cells could be associated with the differential VPA sensitivity and radiation response of cancer cells.

VPA treatment has been reported to provide neuroprotection and improve cognitive function [67, 90]. The neuroprotection and improved cognitive function was due, in part, to inhibition of HDAC and GSK3β, which helped to stabilize antiapoptotic proteins like Bcl-2 in normal HT22 cells and prevent apoptosis ([67] Fig. 1C, 1D & 1E). We also observed that VPA treatment reduced the levels of the pro apoptotic protein Bax (Fig. 1E).

VPA treatment induced a G_2_/M cell cycle arrest in GL261 cells (Fig 3). The accumulation of cells with G_2_/M DNA content is consistent with the induction of mitotic catastrophe as a mode of cell death in irradiated cells. Such effects have been reported for several glioma cells lines treated with VPA [91]. Taken all together, these results suggest that radioprotection by VPA is selective for hippocampal neurons, which could be mediated through the inhibition of HDAC and GSK3β. Although there has been a report that VPA protected rat brains from radiation-induced damage, apoptosis in cerebral cortex cells and long-term blood vessel damage were not quantitated [92].

We used 300 mg/kg VPA in the studies demonstrating radioprotection of normal hippocampal neurons and radiosensitization of glioblastoma in mice. VPA administered at 300 mg/Kg in mice leads to a serum concentration between 265–280 μg/ml [93]. At such serum concentrations, the maximum level of VPA in mouse brains is 17–30 μg/g [94, 95]. The half-life of VPA in mice is 1–3 hours [94], while in humans it is 9–18 hours [96, 97]. In humans the maximum therapeutic dose for epilepsy and mania is 60 mg/kg, which amounts to 750–4000 mg/day [96]. At these doses the therapeutic serum levels are 50–150 μg/ml of which the levels of free VPA are 6–32 μg/ml [98, 99]. The levels of VPA in cerebrospinal fluid are usually similar to the free valproic acid levels [100, 101]. There are differences in VPA metabolism between mice and humans. Thus the findings in mice are difficult to extrapolate directly to humans [102]. Nevertheless, we speculate that the levels of VPA in our studies are comparable to those associated with therapeutic ranges in humans [102].

We found that 300 mg/kg VPA significantly protected mouse hippocampal neurons from radiation damage by attenuating apoptosis (Fig. 1A). Similar neuroprotection by VPA has been observed in animals subjected to ischemia [27, 103], traumatic brain injury [67], and intracerebral hemorrhage [26]. HDAC and/ or GSK3β inhibition has been reported in various studies of animals treated with 300–400 mg/kg of VPA [67, 103, 104].

In preclinical studies treatment with VPA was reported to control the growth of a variety of tumors, including gliomas, neuroblastomas, medulloblastomas and melanomas [22, 49]. VPA's antitumor effects involve the modulation of various cellular and tissue pathways, including cell-cycle arrest, angiogenesis, apoptosis, differentiation, senescence and DNA repair [22]. In the heterotopic GL261 and D54 tumor models, mice treated with VPA were half as likely to develop a tumor with a volume greater than 0.6 cm^3^ than PBS treated mice (Fig. 5B&5E). Tumor growth in mice treated with a combination of VPA and radiation was significantly delayed when compared to radiation alone, VPA alone or PBS treated controls (Fig. 5A & 5B, 6A & 6B). These results indicate that a combination of VPA with irradiation was significantly more effective in controlling tumor growth. VPA treatment alone was also effective in controlling tumor growth in mice and was significantly better than no treatment. More importantly, VPA treatment alone did not enhance tumor growth. These findings suggest that pre-treatment with VPA may significantly improve the response of malignant glioma to radiation therapy.

There are reports indicating that VPA alone or in combination therapy can increase the life span of both tumor bearing mice [105] and humans with brain cancer [48, 56, 58, 60, 61]. In clinical trials, valproic acid was the preferred antiepileptic agent in a combination therapy that utilized radiation and temozolomide [48]. Valproic acid has also been reported to enhance other combination therapies in treating intracranial gliomas in mice [105, 106]. We found similar results while analyzing DCE MRI derived tumor volumes. Mice treated with a combination of VPA and radiation showed significantly reduced tumor growth compared to PBS treated mice (Fig. 6A & 6B). Cox proportional hazards modeling was used to estimate the hazard ratio for overall survival between the treatment levels in the orthotopic intracranial tumor model. We found that mice with no treatment were 11.4 times (*p* = 0.0002) more likely to die than mice treated with combination of VPA and radiation. Mice treated with radiation alone were 4.7 times (*p* = 0.0130) more likely to die than mice treated with combination of VPA and radiation (Fig. 6C). The survival of mice correlated with the tumor volume as seen by DCE MRI (Figs 6A&6B). Overall, these results indicate that VPA in combination with radiation can effectively control tumor growth in the brain, leading to improved survival.

VPA is now drawing attention due to its versatility in improving cognitive sequelae and protecting against various neurological insults, and is emerging as a promising drug for brain cancer treatment [56]. Our current findings on the radioprotective effects of VPA on the hippocampus and radio-sensitization effects of VPA in cancer cells also makes it attractive as a novel therapeutic strategy for the prevention of neurocognitive deficits resulting from cranial irradiation.

## MATERIALS AND METHODS

### Chemicals, cell culture and treatments

Valproic acid was purchased from Sigma. Mouse hippocampal neuronal cells HT22 were obtained from David Schubert (The Salk Institute; La Jolla, CA) and maintained in DMEM with 10% fetal bovine serum (FBS) and 1% penicillin/streptomycin (Life Technologies). Human medulloblastoma Daoy cells were obtained from ATCC and propagated in Eagle's Minimum Essential Medium with 10% FBS, 2 mM L-glutamine, 1.5 g/L sodium bicarbonate, 0.1 mM non-essential amino acids, and 1.0 mM sodium pyruvate. Human glioma D54 and mouse glioma GL261 cell lines were obtained from Dr. Yancey Gillespie (University of Alabama-Birmingham, Birmingham, AL) and maintained in DMEM with Nutrient Mixture F-12 (1:1), 10% FBS, 1% sodium pyruvate and 1% penicillin/streptomycin (Life Technologies). All cells were grown in a 5% CO_2_ incubator at 37°C. Cells were irradiated at a dose rate of 2.5 Gy/min using a RS2000 160 kV X-ray Irradiator with a 0.3 mm copper filter (Rad Source Technologies).

### Histological staining for apoptosis using TUNEL

One-week-old C57BL/6 mice pups were treated with daily i.p. injections of VPA (300 mg/kg) or PBS. On the seventh day of VPA treatment, the pups’ brains were irradiated with 7 Gy. Twenty four hours later, the animals were sacrificed and their brains were harvested and fixed in 10% neutral buffered formalin. The fixed brains were embedded in paraffin and sectioned coronally. Five micron thick sections were placed on Superfrost Gold Plus slides, stained with the DeadEnd™ Colorimetric TUNEL System (Promega) according to the manufacturer's instructions, and counterstained with hematoxylin. For the TUNEL experiments, at least three animals were used in each experimental group. TUNEL positive cells (TPC) were counted using an Olympus BX51 microscope equipped with an Olympus DP26 digital camera. At least three high power fields (HPF) per animal were scored. The average number of TUNEL positive cells per HPF (+/−SD) was calculated.

### Apoptosis assays for cultured cells

Apoptosis was monitored by annexin V-APC/propidium iodide staining using the Apoptosis Detection Kit according to the manufacturer's instructions (BD PharMingen). Briefly, HT22 cells were treated with PBS or VPA (0.6 mM) for seven days, and then irradiated with 4Gy. Sixteen hours post irradiations aliquots of 10^5^ cells were incubated with Annexin V/propidium iodide for 15 minutes at room temperature. The cells were then analyzed by flow cytometry, using a two-color MACS FCM analysis system (Miltenyi Biotech). For each treatment, the average fold-increase of apoptotic cells over control (+/− SD) was calculated.

In separate experiments, the apoptotic nuclei of HT22 cells were counted after staining with 4′, 6-diamidino-2-phenylindole (DAPI). The treated cells were washed with PBS, fixed in 70% ethanol at room temperature for 10 minutes, and stained with DAPI (Vector Labs). The nuclear morphology was monitored using an Olympus BX60 fluorescence microscope equipped with a Retiga 2000R digital camera. Apoptosis was quantified by scoring the percentage of cells with apoptotic nuclear morphology at the single cell level. Condensed or fragmented nuclei were scored as apoptotic; the average percentage of apoptotic cells (+/− SD) was calculated in 5–7 randomly selected HPF.

### Colony formation assay

Equal numbers of GL261 and HT22 cells were plated on 60 mm dishes. After 4–5 h, cells were treated with either PBS or 0.6 mM VPA for seven days. Colonies were allowed to form for 10 days, after which cells were fixed with 70% ethanol and stained with 1% methylene blue. Colonies having greater than 50 cells were counted under a microscope and the results plotted. Experiments were repeated in triplicate and standard errors were calculated.

### Clonogenic survival assay

Defined numbers of cells were plated and allowed to attach for 5 hours and then irradiated with 0, 2, 4, 6 or 8 Gy. After 7–10-day incubation, plates were fixed with 70% ethanol and stained with 1% methylene blue. Colonies consisting of more than 50 cells were counted by viewing the plates under a StemiVD4 dissecting microscope (Zeiss). The survival fractions were calculated as (number of colonies/number of cells plated)/(number of colonies for corresponding control/number of control cells plated). The dose modifying factors at 10% survival (DMF_10_) were calculated from the survival curves by taking the ratio of the dose of IR that reduced survival to 10% divided by the dose of IR that reduced survival to 10% in the presence VPA.

### Immunoblotting

Total protein was extracted from treated cells using the M-PER mammalian protein extraction reagent (Pierce). Protein concentration was determined using the BCA Reagent (Pierce). Protein extracts were analyzed using antibodies for the detection of phospho-GSK3β^Ser-9^, GSK3β, β-catenin, PARP, Bcl-2 (Cell Signaling Technologies), Acetyl H4 (Millipore) and Bax (Santa Cruz). Antibody against actin (Sigma) was used to normalize protein loading in each lane. Bands were visualized using the Western Lightning Chemiluminescence Plus detection system (PerkinElmer), according to the manufacturer's protocol.

### Colorimetric cell proliferation assay

Cell proliferation was determined using Cell Titer 96 aqueous non-radioactive cell proliferation assay reagent (Promega). The assay was performed following the manufacturer's protocol. Briefly, cells were treated with PBS or 0.6 mM VPA for seven days and equal numbers of treated HT22 (1000 cells), GL261 (2000 cells), D54 (2000 cells) and Daoy (2000 cells) cells were plated in 96-well plates. The cells plated in the 96 well plates had the respective drug or vehicle control. Cell viability was determined after incubation for 96 h by measuring the absorbance at 490 nm with a Countess II L plate reader (Life Technologies). The cells in 96-well plate were visually scanned in an inverted microscope AE30 (Motic) after 96 h of incubation, to ensure that cells were not overly confluent in some experimental arms before performing the proliferation assay. Experiments were performed in triplicate, and both average fold changes relative to controls and standard errors were calculated.

### Cell cycle analysis by flow cytometry

HT22 and GL261 cells were treated with PBS or 0.6 mM VPA for seven days, and then irradiated with 4Gy. Twenty-four hours post irradiation; treated cells were washed with PBS, fixed in 70% ethanol, and incubated overnight at −20°C. Cells were pelleted and resuspended in PBS with DNAse-free RNAse (20 μg/ml) and incubated at 37°C for 30 min. The cells were then stained with propidium iodide (50 μg/ml) at room temperature for 15 min and analyzed by flow cytometry. Cell cycle analysis was performed using Modfit LT 3.0 software. The average fractions of cells in the G_1_, G_0_, G_2_-M and S phases of the cell cycle were determined and the standard error was calculated from three separate experiments.

### Mice and treatments

C57BL/6 and Nu/Nu mice (Harlan) were used for animal studies. Animal procedures were approved by the Institutional Animal Care and Use Committee (IACUC) at Washington University School of Medicine in St. Louis. Mice were irradiated at a dose rate of 1 Gy/min using RS2000 160 kV X-ray Irradiator with a 0.3 mm copper filter (Rad Source Technologies). Mice were anesthetized with 2.5% isoflurane delivered using oxygen to immobilize the mice. Only the intended organ or the tumor was exposed and the rest was shielded with lead during irradiation.

### Tumor growth delay

One million GL261 or D54 cells were injected into the right flank of each C57BL/6 mouse. Once tumors were palpable, mice were stratified into four treatment groups of eight each representing similar distributions of tumor sizes. Tumors from two groups of mice were irradiated with 2 Gy fractions daily for five consecutive days for a total of 10 Gy. These mice received either 300 mg/kg VPA or PBS as vehicle control by intraperitoneal injection (i.p.), 60 min prior to each irradiation. Two groups of non-irradiated mice received either 300 mg/kg VPA or PBS alone by i.p. injection daily for five days as well. Tumor volumes in the mouse hind limbs were measured using calipers. The mean tumor volume and standard error were calculated for each treatment group.

### Intracranial tumors

Orthotopic intracranial tumors were induced by stereotactically injecting 5 × 10^4^ GL261-FG cells into the brains of six-week-old nude mice at a position 2 mm lateral to the bregma at a depth of 3 mm. The GL261-FG cells were transduced with a lentivirus for stable expression of both firefly luciferase and green fluorescent protein. Ten days after injecting the GL261-FG cells, the presence of tumor was confirmed by bioluminescence imaging (BLI) [107]. The mice were then stratified into four treatment groups of nine, each representing similar distributions of tumor sizes. Tumors from two groups of mice were irradiated daily with 2Gy for five consecutive days for a total of 10 Gy. Mice were anesthetized and irradiated in a holder designed to irradiate the tumor region in the head only and shield the rest of the body with lead during irradiation. These mice received either 300 mg/kg VPA or PBS as vehicle control i.p., 60 min prior to irradiation. Two groups of non-irradiated mice also received either 300 mg/kg VPA or PBS alone i.p., daily for five days as well. Over the course of 30 days, mice were weighed daily and monitored closely for the signs of pre-morbid state. These signs included hypoactivity, shallow, rapid and/or labored breathing, failure to groom, failure to respond to stimuli, hunched posture, dehydration and weight loss. When any of these signs were present, mice were euthanized. Surviving animals were euthanized at the end of the experiment (45 days after tumor implantation). Survival was calculated using Kaplan-Meyer analysis.

### MRI imaging of intracranial tumors

Magnetic resonance images were collected using an Oxford Instruments 4.7-T magnet (40 cm, clear bore) equipped with 21-cm inner diameter, actively shielded Agilent/Magnex (Yarnton) gradient coils (maximum gradient 28 G/cm; rise time ~650 μs) driven by model A-240 amplifiers (Oy International Electric Company) The magnet/gradients were interfaced with an Agilent/Varian Direct Drive console, and data were collected using a 1.5-cm OD surface coil (receive) and a 5-cm ID Helmholtz coil (transmit) actively decoupled coil pair as described [108]. Before the imaging experiments, mice were anesthetized with isoflurane/O_2_ [2–3% (v/v)], and maintained on isoflurane/O_2_ [1% (v/v)] throughout the experiments. Animal body temperature was maintained at 37°C using a pad heated with warm circulating water. Mice were injected i.p. with 500 μl MultiHance contrast agent (Bracco Imaging), diluted 1:10 in sterile saline, 15 minutes prior to being placed in the magnet. T_1_-weighted, spin-echo multi-slice transaxial images were collected with T_R_ = 0.65 s, T_E_ = 0.02 s, FOV = 1.5 × 1.5 cm^2^, slice thickness = 0.5 mm.

### Statistical analyses

The means and standard deviations (SD) of each treatment group were calculated for all experiments. The number of samples is indicated in the description of each experiment. Kaplan-Meier and Cox Proportional Hazards models were used to analyze survival data, while non-parametric Kruskal-Wallis analysis was used to compare treatment effectiveness on tumor growth delay. All pair-wise comparisons between treatment groups were adjusted using Tukey's multiple comparison method. A *p*-value of < 0.05 was considered statistically significant.

## SUPPLEMENTARY FIGURES


