# Supplementary material for: Valproic acid enhances the efficacy of radiation therapy by protecting normal hippocampal neurons and sensitizing malignant glioblastoma cells
Source: Oncotarget. 2015 Sep 16;6(33):35004–22. doi: 10.18632/oncotarget.5253 (PMC4741505; doi:10.18632/oncotarget.5253)
Supplement: Supplementary file 1 [file oncotarget-06-35004-s001.pdf]

SUPPLEMENTARY FIGURES

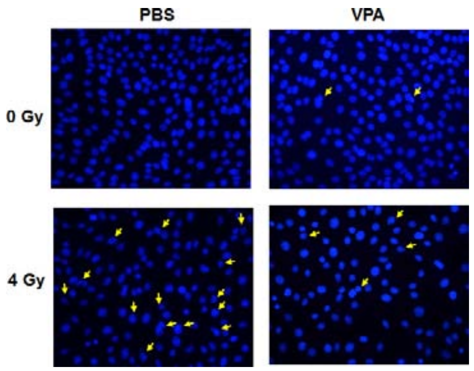

Supplementary Figure S1: Valproic acid attenuates radiation-induced apoptosis in HT-22 cells. HT-22 cells were treated with PBS or 0.6 mM Valproic acid for 7 days prior to irradiation with 4 Gy. 24 h after irradiation, cells were fixed and stained with DAPI. Shown are the micrographs after DAPI staining. Apoptotic cells indicated by arrows were counted in eight randomly selected HPF.

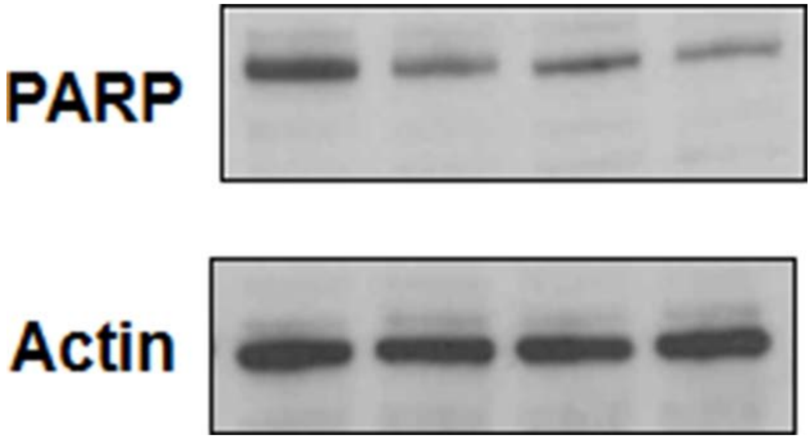

Supplementary Figure S2: PARP cleavage is not detected in irradiated HT-22. HT22 cells were treated with PBS or 0.6 mM VPA for 7 days prior to irradiation with 4 Gy. Whole cell extracts were immunobloted to determine PARP cleavage. Actin was used to normalize the protein loading in each lane.
